# Problems of middle ear and hearing in cleft children

**DOI:** 10.4103/0970-0358.57198

**Published:** 2009-10

**Authors:** Ramesh Kumar Sharma, Vipul Nanda

**Affiliations:** Department of Plastic Surgery, Postgraduate Institute of Medical Education and Research, Chandigarh-160 012, India

**Keywords:** Cleft lip and palate, Hearing loss, Middle ear infection

## Abstract

The hearing loss in a cleft patient is a well known complication, but generally gets ignored. These children continue to have recurrent otitis media with effusion that affects the hearing abilities. Unfortunatley the middle ear function may not improve with palatoplasty.Cleft palate teams need to follow up all such children beginning at birth and going into adulthood, decades after a ‘successful’ palate repair. These patients should have careful otological and audiological surveillance with appropriate interventions whenever required. The review article discusses the current status of hearing management in patients with cleft palate.

## INTRODUCTION

Hearing loss is a well-known complication of cleft palate but the magnitude of this problem is not generally appreciated A lot of attention has been paid to the development of a competent velopharyngeal port and normal facial development in these children. Unfortunately, this concern with the production of normal speech and the prevention of facial deformity has diverted attention from a common but unfortunately ignored complication of hearing loss.

Middle ear disease is common in childhood. Seventy-one percent of all children have at least one episode of otitis media by the age of three years.[1] In contrast children with cleft palate have been reported to have recurrent acute otits media or otitis media with effusion.[2] Dhillon describes a 97% incidence of otitis media with effusion in children with cleft palate, less than 24 months of age.[3]

It has been found that the incidence of hearing problems in cleft lip alone is the same as in the controlled population. The incidence, however, increases sharply when there is associated submucous cleft palate in this group.[1]

Lou *et al*.[4] observed an incidence of 90% in Caucasian patients whereas in Chinese patients the incidence has been reported to be only 23%. In the Japanese population also, a similar lower incidence of 69% has been found[5] (as quoted by Muntz HR in Fac Plast Surg 1993;9:177-180). In a recent study from India[6] 43 patients with operated cleft palate were assessed in a speech camp; three-fourths of the patients (75%) had hearing problems.

Although one would expect that the middle ear function would improve after the palatoplasty, the available reports suggest that there is no improvement in the incidence.[7,8]

## MIDDLE EAR ANATOMY

The middle ear cavity is located in the mastoid process of the temporal bone. The cavity extends from the tympanic membrane to the inner ear. It is approximately two cubic centimetres in volume and is lined with mucous membrane. The middle ear cavity is actually an extension of the nasopharynx via the Eustachian tube.

### Eustachian Tube

The Eustachian tube acts as an air pressure equalizer and ventilates the middle ear. Normally, the tube is closed but opens while chewing or swallowing. When the Eustachian tube opens, the air pressure between the outer and middle ear is equalized. The transmission of sound through the eardrum is optimal when the air pressure is equalized between the outer and middle ear. When the air pressure between the outer and middle ear is unequal, the eardrum is forced outward or inward causing discomfort and the ability of the eardrum to transmit sound is reduced.

### Ossicles

The middle ear is connected and transmits sound to the inner ear via the ossicular chain. The ossicular chain amplifies a signal approximately 25 decibels as it transfers signals from the tympanic membrane to the inner ear. The ossicular chain consists of the three smallest bones in the body: the malleus, incus, and stapes. The malleus is attached to the tympanic membrane. The footplate of the stapes inserts into the oval window of the inner ear. The incus is between the malleus and the stapes. Attached to the ossicular chain are two muscles, the stapedius and tensor tympani muscles. These muscles contract to protect the inner ear by reducing the intensity of sound transmission to the inner ear from external sounds and vocal transmission.

### Muscles

The middle ear is part of a functional system composed of the nasopharynx and the Eustachian tube (anteriorly) and the mastoid air cells (posteriorly). The only active muscle that opens the Eustachian tube is the tensor veli palatini, which promotes ventilation of the middle ear. An understanding of the anatomy and physiology of the system can aid the clinician in understanding the role of Eustachian tube dysfunction in the cause and pathogenesis of middle ear disease. The tendon of the tensor veli palatini hooks around the hamulus of the pterygoid plate and the aponeurosis of the muscle inserts along the posterior border of the hard palate. The muscle originates partially on the cartilaginous border of the auditory tubes. The function of the tensor veli palatini, similar to tensor tympani with which it shares similar innervation, is to improve the ventilation and drainage of the auditory tubes.

In a cleft of the palate, the aponeurosis of the tensor veli palatini, instead of attaching along the posterior border of the hard palate, is attached along the bony cleft edges. This abnormality in the tensor veli palatini decreases its effectiveness as a Eustachian tube ‘opener’ and increases the incidence of middle ear effusion and middle ear infection.

## PATHOGENESIS OF MIDDLE EAR PROBLEMS IN CLEFT PALATE (AFTER MASTERS *et al.)[9]*

### Mechanical

The abnormal reflux of food and fluid into the nasal cavity can set up chronic inflammatory changes around the Eustachian orifices with oedema, hypertrophy of adenoid pads, low-grade obstruction, and secondary middle ear disease.

### Infection

The break in the mechanical barrier between the mouth and nasopharynx with its concomitant chronic inflammatory change alters the bacterial flora of the region to permit overgrowth of predominately pathogenic bacteria. This results in otitis media.

### Dynamic

The dynamic factor in Eustachian tube and middle ear physiology depends upon the intact anatomy of Eustachian apparatus and its extrinsic musculature. In the normal resting phase, the Eustachian orifices are closed, opening widely with yawning and swallowing, and also opening slightly with normal speech. This opening of the Eustachian orifice permits aeration and pressure equalization as well as relief of obstruction in the Eustachian canal, and is dependent upon the action of the intact tensor and levator palatini muscles. The rapid motion of these muscles, particularly in speech, may produce almost a milking action in the cartilaginous portion of the Eustachian tube. Thus, the dilation of the orifices dependupon muscles which are not intact in the child with a congenital cleft of the palate. This dynamic function of the tensor and levator muscles is well known in speech as velopharyngeal closure largely depends upon their contraction, but the secondary opening and closing of the Eustachian orifices may be of equal importance in the prevention of hearing loss.

At rest the Eustachian tube is closed. When it is opened it ventilates the middle ear, releases mucus and equalizes pressure differentials that are created by gaseous absorption and environmental pressure changes. In children with an unrepaired cleft palate, the tensor muscle fibres do not have a normal course and midline palatal insertion and, therefore, lack the anchorage to effectively open the Eustachian tube. In this situation, when gases are absorbed by the mucous membrane of the middle ear they are not replaced, resulting in negative pressure. Sustained negative pressure results in a retracted tympanic membrane and eventual secretion of fluid into the middle ear space from the mucous membrane.

## ANALYSIS OF CRANIOFACIAL SKELETON IN CLEFT CHILDREN WITH OTITIS MEDIA WITH EFFUSION

Kemaloglu[10] *et al*. have analyzed the craniofacial skeleton and have suggested that there are many factors in the skeleton that predispose these children to Otitis Media with Effusion(OME) They inferred that small dimensions of the posterior cranial base (spheno-occipital bone) and backward and upward position of the maxilla were associated with tendency to OME in clefts. In addition, mastoid depth and height were also shorter in cleft cases than normal subjects. On the other hand, a small tendency to recurrent upper airway infection (RUAI) was observed in cleft cases with OME. Further, it was found that the following differences in the mastoid-middle ear-Eustachian tube (M-ME-ET) system were associated with a tendency to OME in unilateral cleft lip and palate UCLP cases: more horizontal ET in relation to the posterior cranial base; short bony ET; short height and antero-posterior depth of the mastoid air cell system.

## DISCUSSION

As clinicians we are faced with the following research questions:

### Does palate repair reduce incidence of middle ear pathology?

Little is known about the natural history of patients with a cleft palate who have not undergone a palatoplasty or any intervention for their otologic complaints - the ‘no palatoplasty, no'ear nose thoroat (ENT) care group’. Studies from India and China on patients with unrepaired cleft palate show that a significant percentage of such individuals demonstrated a hearing loss and abnormal tympanometry persisting into adolescence and adulthood.[11–13] A study from Turkey of the audiological profile of children whose palates were repaired during the first two years of life and who had no access to any ENT care showed the prevalence of middle ear disease to be much lower than the ‘no palatoplasty, no ENT care’ group.[14] This comparative data proves palate repair to be an independent variable in reducing the prevalence of middle ear pathology. We may extrapolate this to build up the case for the beneficial effect of early palate repair vis-a-vis middle ear pathology.

### Does the type of repair have any bearing on the middle ear pathology?

There is no evidence that a particular technique is superior with respect to preserving middle ear physiology. Cutting's technique reports the use of tensor tenopexy of the tendon of the tensor veli palatini at the hamulus prior to the formal tenotomy.[15] This is to allow the cut tensor to continue functioning and aid in Eustachian tube opening. The results of this procedure have however, has not yet been published. A recent report from Smith *et al.* suggests that children who underwent cleft repair with Furlow's double opposing z plasty technique had fewer ear tubes placed postoperatively than patients who had traditional repair.[16]

### Hearing levels and age in cleft palate patients

In an interesting study from Croatia by Handzik-Cuk *et al*.,[17] operated patients with isolated cleft palate showed greater improvement in hearing level with age than patients with UCLP and bilateral cleft lip and palate BCLP; as adults they showed the lowest incidence of ears with hearing level of less than 40 dB, and the highest frequency of ears with hearing levels of 11-20 dB. Patients with BCLP had a higher frequency of ears with a hearing level of 21-40 dB during early childhood and adult age than patients with isolated cleft palate ICP. Patients with UCLP and BCLP showed a slower decrease with age in the frequency of ears with hearing loss than patients with ICP; the hearing level in patients with UCLP and BCLP improved only in groups with hearing levels of 21-40 dB, while those with hearing levels above 40 dB showed no significant improvement with age.

### What are the recommendations for management of the middle ear?

The tendency to develop OME is reduced but not reversed by early palatal surgery. Children should have careful Ear Nose and Throat (ENT) and audiological surveillance even after the palate has been repaired. Management strategies for otitis media in cleft palate children vary among different teams internationally. Ventilation tube (grommet) insertion may occur routinely at the time of palatoplasty or selectively on a separate occasion if symptomatic middle ear disease develops. Some teams feel that the frequency of OME in cleft palate is such as to warrant early tympanostomy tubes and report good audiological outcomes.[18]

OME occurs in the majority of patients with cleft palate. In these patients middle ear disease persists longer and leads to higher incidence of conductive hearing losses, language disability and cholesteatoma formation as compared to their peers.[19]

Although there is a universal consensus about the occurrence of otitis media in children with cleft palate before cleft repair, controversy continues regarding the recovery of Eustachian tube function and level of hearing loss in patients after cleft palate repair. Early and aggressive ventilation tube placement is the standard care of patients with cleft palate in many countries, and several reports in the literature discuss the outcomes of these patients.[20] Despite liberal use of ventilation tubes, debate about conservative versus aggressive treatment of the middle ear disease continues.

Although early and routine placement of ventilation tubes to alleviate hearing problems is not universally accepted, it is a part of cleft palate care in many countries. At follow-up examinations, Robinson *et al*.[21] found more abnormal otologic conditions in children who had tubes inserted, some of which were directly attributable to the presence of the tubes. Insertion and reinsertion of ventilation tubes may have a negative impact on middle ear structure and hearing.

A number of investigations have found that closure of cleft palate significantly reduced the prevalence of audiological problems. Gopalakrishna *et al*.[11] indicated that Eustachian tube function remained poor in patients with untreated cleft palate. Desai[22] suggested that if palate repair is done within the first four weeks of life, chances of middle ear disease should decrease theoretically as till four weeks the Eustachian tube functions normally even in children born with cleft palate.

The alternative approach is to offer tympanostomy tubes only when there is overt evidence of OME. A recent study from New Zealand has recommended the placement of ventilation tubes only in patients selected on the basis of symptomatic infection or significant hearing loss.[23] The provision of hearing aids should be considered in suitable cases.[19] The tendency to develop OME is reduced but not reversed by early palatal surgery. Children should have careful ENT and audiological surveillance even after the palate has been repaired. There is currently insufficient evidence on which to base the clinical practice of early routine grommet placement in children with cleft palate.[20]

Goudy *et al*.[24] in a recent review have concluded that myringotomy tube insertion for treatment of otitis media with effusion and/or conductive hearing loss is common practice, although its usefulness is questioned. Eustachian tube dysfunction resolves after palate repair in 50% or more, and resolves in most patients by the age of five. Thought should be given to the use of long-term ventilation tubes in patients requiring multiple sets, due to the association of hearing loss with greater than three or four tube insertions. In patients with persistent conductive hearing loss, 44% were bilateral, warranting close observation and treatment. Lifelong otologic evaluation is recommended due to the 5.9% risk of cholesteatoma.

## CONCLUSIONS

Cleft palate teams need to follow up all such children beginning at birth and going into adulthood, decades after a ‘successful’ palate repair. These patients should have careful otological and audiological surveillance with intervention as appropriate.
